# Effects of Novel Inverted Rocker Orthoses for First Metatarsophalangeal Joint on Gastrocnemius Muscle Electromyographic Activity during Running: A Cross-Sectional Pilot Study

**DOI:** 10.3390/s20113205

**Published:** 2020-06-05

**Authors:** Rubén Sánchez-Gómez, Carlos Romero-Morales, Álvaro Gómez-Carrión, Blanca De-la-Cruz-Torres, Ignacio Zaragoza-García, Pekka Anttila, Matti Kantola, Ismael Ortuño-Soriano

**Affiliations:** 1 Nursing Department, Faculty of Nursing, Physiotherapy and Podiatry, Universidad Complutense de Madrid, 28040 Madrid, Spain; rusanc02@ucm.es (R.S.-G.); alvaroalcore@hotmail.com (Á.G.-C.); izaragoz@ucm.es (I.Z.-G.); iortunos@ucm.es (I.O.-S.); 2 Faculty of Sport Sciences, Universidad Europea de Madrid, Villaviciosa de Odón, 28670 Madrid, Spain; 3 Department of Physiotherapy, University of Seville, c/Avicena, s/n, 41009 Seville, Spain; bcruz@us.es; 4 Applied Science of Metropolia Univesity, Podiatry Department, 01600 Helsinki, Finland; pekka.anttila@metropolia.fi (P.A.); Matti.Kantola@metropolia.fi (M.K.)

**Keywords:** triceps surae, first metatarsophalangeal joint, surface electromyography

## Abstract

Background: The mobility of the first metatarsophalangeal joint (I MPTJ) has been related to the proper windlass mechanism and the triceps surae during the heel-off phase of running gait; the orthopedic treatment of the I MPTJ restriction has been made with typical Morton extension orthoses (TMEO). Nowadays it is unclear what effects TMEO or the novel inverted rocker orthoses (NIRO) have on the EMG activity of triceps surae during running. Objective: To compare the TMEO effects versus NIRO on EMG triceps surae on medialis and lateralis gastrocnemius activity during running. Study design: A cross-sectional pilot study. Methods: 21 healthy, recreational runners were enrolled in the present research (mean age 31.41 ± 4.33) to run on a treadmill at 9 km/h using aleatory NIRO of 6 mm, NIRO of 8 mm, TMEO of 6 mm, TMEO of 8 mm, and sports shoes only (SO), while the muscular EMG of medial and lateral gastrocnemius activity during 30 s was recorded. Statistical intraclass correlation coefficient (ICC) to test reliability was calculated and the Wilcoxon test of all five different situations were tested. Results: The reliability of values was almost perfect. Data showed that the gastrocnemius lateralis increased its EMG activity between SO vs. NIRO-8 mm (22.27 ± 2.51 vs. 25.96 ± 4.68 mV, *p* < 0.05) and SO vs. TMEO-6mm (22.27 ± 2.51 vs. 24.72 ± 5.08 mV, *p* < 0.05). Regarding gastrocnemius medialis, values showed an EMG notable increase in activity between SO vs. NIRO-6mm (22.93 ± 2.1 vs. 26.44 ± 3.63, *p* < 0.001), vs. NIRO-8mm (28.89 ± 3.6, *p* < 0.001), and vs. TMEO-6mm (25.12 ± 3.51, *p* < 0.05). Conclusions: Both TMEO and NIRO have shown an increased EMG of the lateralis and medialis gastrocnemius muscles activity during a full running cycle gait. Clinicians should take into account the present evidence when they want to treat I MTPJ restriction with orthoses, and consider the inherent triceps surae muscular cost relative to running economy.

## 1. Introduction

Coterill [1] was the first author who described painful osteoarthritis (OA) of the first metatarsophalangeal joint (IMTPJ), which is known as hallux rigidus (HR). HR is the last stage of the IMTPJ degeneration, with functional hallux limitus [2] (FHL) at the beginning of the pathological progress [3]. Joint disease is thought to be caused by repetitive impacts on the dorsal aspect of the base of the proximal phalanx of the hallux by the first metatarsal head during the propulsion phase of gait and running in feet with multifactorial biomechanical and/or structural deficits [4]. The limitation of IMTPJ has been linked to gait problems [5] and its consequences on ankle, knee, hip, or low back during running [6].

The treatment of this injury has been addressed in several conservative non-surgical and surgical ways. Non-surgical management is valid to treat HR in the earliest stages [7,8] and includes ultrasound therapy, infiltrative drugs, shoe modifications, hallux bandages, manual mobilization, flexor strengthening, and orthoses to improve the joint problems. There are a few references on treatment of OA using plantar insoles in HR and FHL. Traditional Morton’s extensions are orthoses with a flat light modification under the first ray that has been used to treat HR [9,10,11] to avoid the impact between the proximal phalanx and first metatarsal bones. This opens the IMTPJ dorsally but restricts its dorsiflexion movement, while rocker-sole footwear modifications have shown a reduction in the peak pressure under the IMTPJ. This decreases the average gait cycle that is spent in the stance phase [12] and increases muscle activity of the lower limb [13]. However, there is no reference to either the inverted rocker-sole orthoses effects or the effect of footwear modifications on muscle activity during running.

On the other hand, running economy (RE) has been described as the oxygen cost of running at a given speed in every case [14] and factors such as biomechanics and muscular fatigue can influence the RE [15]. Additionally, barefoot running has shown differences in biomechanical behaviour [16] and muscular responses [17,18] when it is compared with classical running shoes. Compared to fatigue, strength training added to a normal training program for distance running can improve RE between 2% and 8%. An increase in muscle mass training programs around the proximal region of the lower limb, such as quadriceps or hamstring [19], or around the distal regions, such as the triceps surae [20] with plantarflexion and dorsiflexion ankle exercises, has shown some benefits on RE. Accordingly, triceps surae and its relationship with the windlass mechanism [21] in the propulsion phase of gait and running has been reported to provide between 8% and 17% of the elastic energy that is needed for the heel-off phase [22,23] toward a suitable IMTPJ dorsiflexion [24,25]. However, the electromyography (EMG) effects in the triceps surae with limited dorsiflexion of the IMTPJ that is induced by any orthotic dorsiflexion restriction has never been studied. Understanding the EMG activity of this muscle will allow us to understand if the subjects could be increasing their energy cost during running, which is very important for an efficient RE [19]. However, no previous research has studied the effect of a novel inverted rocker orthoses (NIRO) on the EMG activity of the triceps surae compared to traditional Morton’s extension orthoses (TMEO) during running in the healthy population. Because of the restricting IMTPJ effect of TMEO and its influence on the windlass mechanism that is linked with the triceps surae [24,25], we hypothesized that TMEO (6 mm and 8 mm) may increase the EMG activity of the gastrocnemius medialis and lateralis muscles compared to the shoe only (SO) condition during running activity; in addition, regarding previous muscular activity changes that are reported with classical rocker soles [13], we hypothesized that NIRO (6 mm and 8 mm) may reduce the EMG of gastrocnemius medialis and lateralis compared to TMEO (6 mm and 8 mm), and this may increase EMG compared to SO in healthy people during running activity.

## 2. Materials and Methods

The public institutional review board at Virgen Macarena-Virgen del Rocío hospitals, reviewed and approved the present study (certificate number f7f4a6567676d7ba7163bce0d15e7f98c9f33354). Ethical and human criteria were followed according to the Declaration of Helsinki, and signed informed consent was obtained from all subjects.

### 2.1. Design and Sample Size

The statistics unit at the Spanish public university used software to assess the suitable sample size to perform this cross-sectional observational study and to study the difference in the EMG changes in the gastrocnemius medialis and lateralis muscles between SO, NIRO 6 mm, NIRO 8 mm, TMEO 6 mm, and TMEO 8 mm groups during running. Previous data on the triceps surae showed 7.0 ± 0.6 millivolts (mV) wearing 9-mm heel lifts compared to 4.9 ± 0.6 mV wearing typical shoes [26]. Taking into account a statistical power of 80%, β = 20%, a 95% confidence interval (CI), and α = 0.05, 30 subjects were needed to complete the study. Considering the typical loss of 20% subjects, 24 participants were recruited. However, three individuals were excluded from the study because they felt pain and discomfort during the EMG assessment. Reporting of Observational Studies in Epidemiology (STROBE) [27] criteria and a randomly consecutive sampling technique were followed to develop the present research.

### 2.2. Subjects

The following inclusion criteria were used to select the participants: (1) healthy participants, between 18 and 30 years old; (2) recreational runners with 3–4 h of training per week with more than 1 year of experience; (3) neutral foot posture index (FPI) with values between 0 and +5 points according to a validity tool [28]; and (4) no injuries or pain at the time of the test. The exclusion criteria were as follows: (1) any lower limb injury during the last 6 months; (2) less movement in either foot joint than what is required to perform the optimal biomechanics according to normal values [29,30]; or (3) under the influence of any drugs effects at the time of the measurements. Body mass index (BMI) was taken into account to select a homogeneous sample, using Quetelet’s equation as follows: BMI = weight (kg)/height (m^2^) [31].

### 2.3. Instrumentation and Assessments

Neurotrac^®^ Simplex Plus (Verity Medical Ltd., Braishfield, UK) EMG electronic device with a USB-Bluetooth [32] was used to study the triceps surae activity during the running test. The recording range on the device was 0.2 mV to 2000 mV, with a sensitivity of 0.1 mV RMS, 10 m of free wireless (Bluetooth) connection range and an accuracy of 4% of the reading from mV +/− 0.3 mV to 200 Hz, with a bandpass filter of 18 Hz +/− 4 Hz to 370 Hz +/− 10% for readings below 235 mV. The signal was assessed using self-adhesive circular surface electrodes that were 30 mm in diameter and made of high-quality hydrogel and conductive carbon film to detect the electrical action of the muscle fibers. The signal from each electrode was captured by the receiver module and filtered automatically by the Neurotrac^®^ software (Verity Medical Ltd., Braishfield, UK). It was sent by a unidirectional radioelectric secure connection to the computer and it was digitally transformed by the software to generate activity patterns data for each electrode.

### 2.4. Materials

**Figure 1:**
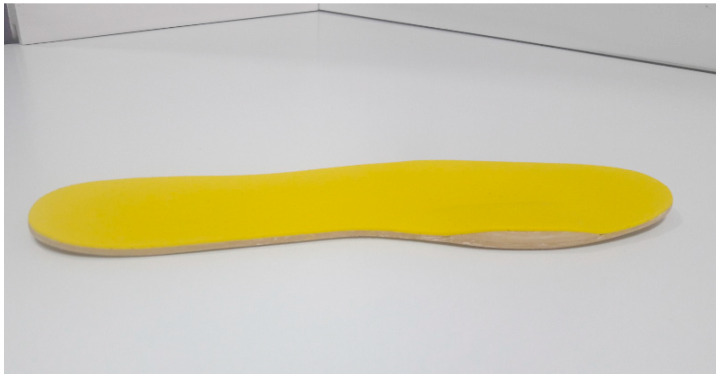
Novel inverted rocker orthotic (NIRO).

**Figure 2:**
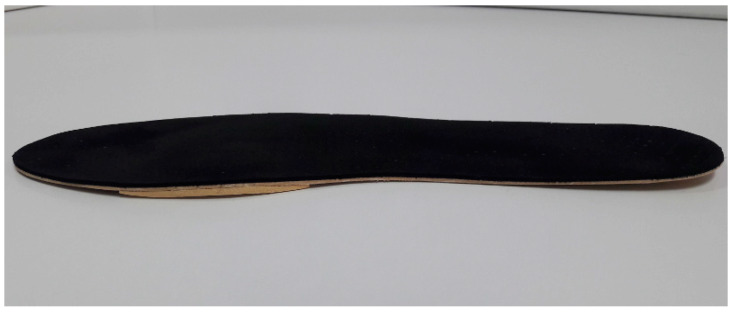
Typical Morton’s extension orthotic (TMEO).

NIRO was made using a flat sheet of ethylene-vinyl acetate (EVA) with a semi-rigid density that was 3 mm thick, without any orthotic element that could interface with normal biomechanical behaviour of the foot. NIRO had an inverted rocker composed of EVA medium that was 5 cm long, 2 cm wide, and 6 mm thick. Its proximal and distal edges were smoothly polished, and it was placed on the IMTPJ. The whole orthotic was covered with an EVA soft layer that was 1 mm thick (Figure 1). The TMEO was made with the same flat sheet of semi-rigid EVA that was 3 mm thick without any orthotic element and with a rectangular flat piece of EVA medium (6 mm thick) that was placed under the IMTPJ area and it was covered with an EVA soft layer that was 1 mm thick (Figure 2). The neutral SOs were “New Feel PW 100M medium grey” (ref. number: 2018022). NIRO and TMEO were made in an external orthopedic laboratory that was blinded to the study protocol.

A flat sheet of ethylene-vinyl acetate (EVA) with an inverted rocker piece of EVA medium 6 mm thick under IMTPJ (bulked raised shape) covered with a yellow EVA soft layer that was 1 mm thick.

A flat sheet of ethylene-vinyl acetate (EVA) with a rectangular flat piece of EVA medium 6 mm thick under IMTPJ covered with a black EVA soft layer that was 1 mm thick.

### 2.5. Procedure

**Figure 3:**
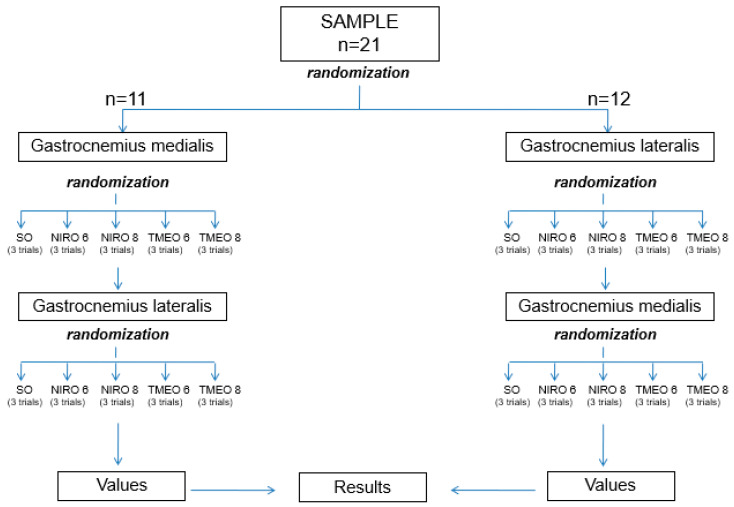
Randomized flow chart. Abbreviations: SO = shoe only; NIRO = novel inverted rocker orthoses; and TMEO = traditional Morton extension’s orthoses.

The podiatric clinician researcher (RSG) performed a physical assessment of the subjects and applied the eligibility criteria. To visualize the muscle belly, each subject was asked to perform plantarflexion of the ankle joint for a few seconds. The surface electrodes were then placed longitudinally onto the most prominent bulge of the gastrocnemius medialis and lateralis, based on the “European recommendations for surface EMG” [33]. The subjects were then asked to stand on one leg in the tip-toe position using their dominant foot for 5 s to set the maximal voluntary contractions that were needed in the strongest limb to calibrate the software and to normalize EMG data amplitudes for each test [34]. This was followed by acclimatization of subjects to a motorized treadmill at 5.17 km/h for 3 min [17]. The participants were divided randomly in gastrocnemius lateralis or medialis group by choosing a sealed envelope that assigned them to one group or another to begin the test; after that, they selected one of the five sealed envelopes with each of the five different conditions of the study (SO, NIRO 6 mm, NIRO 8 mm, TMEO 6 mm, TMEO 8 mm) to set randomly the order of the test. The 11 subjects who began with medialis gastrocnemius assessments, did the lateralis test following the same randomized protocol for each of the five different conditions and vice versa for the 12 participants who began with the lateralis test (Figure 3). Three running trials at 9 km/h [35] under five different conditions (SO, NIRO 6 mm, NIRO 8 mm, TMEO 6 mm, and TMEO 8 mm) on the same day were randomly performed. The duration of each trial was 1 min. For each subject, the mean EMG muscle activity pattern [36] of the gastrocnemius medialis of the dominant leg was recorded during the last 30 s of each 1-min trial, which was performed three times, leaving 5 min of rest between each test [37]. To avoid a potential imbalance, the same condition was added to contralateral foot. The same protocol was performed to assess another gastrocnemius EMG activity pattern. Subjects were blinded to which of the five random conditions that they were wearing, and the results were used to test the hypothesis.

### 2.6. Statistical Analysis

To test for reliability in the present research, within-day trial-to-trial intraclass correlation coefficient (ICC) and the standard error of measurement (SEM) were calculated for the subjects under the five conditions for each muscle during the running test [14]. According to Landis and Koch [38], coefficients of ICC that were lower than 0.20 indicated a slight agreement, 0.20–0.40 indicated fair reliability, 0.41–0.60 indicated moderate reliability, 0.61–0.80 indicated substantial reliability, and 0.81–1.00 indicated almost perfect reliability. The authors considered coefficients of ≥0.81 to be appropriate to consider the results of the study as valid. SEM assessed the minimal detectable change (MDC) for all measurements. This is known as reliable change index (RCI), and it was used to determine the clinical significance of the data [39]. The Shapiro–Wilks test was used to assess the normality of the sample, and normal a distribution was present if *p* >0.05. Demographic values were presented as the mean and standard deviation (±SD). The *p*-values for multiple comparisons were corrected with a non-parametric paired Friedman test to prove that all SOs, NIROs, and TMEOs conditions were different between them. The Wilcoxon test with Bonferroni’s correction was performed to analyze differences between the five different conditions, indicating statistically significant differences when *p* < 0.05 with a 95% CI. All the values that were generated using NeuroTrac^®^ software were loaded into Excel^®^ template (Windows^®^ 97–2003), and they were analyzed using SPSS version 19.0 (SPSS Science, Chicago, IL, USA).

## 3. Results

**Figure 4:**
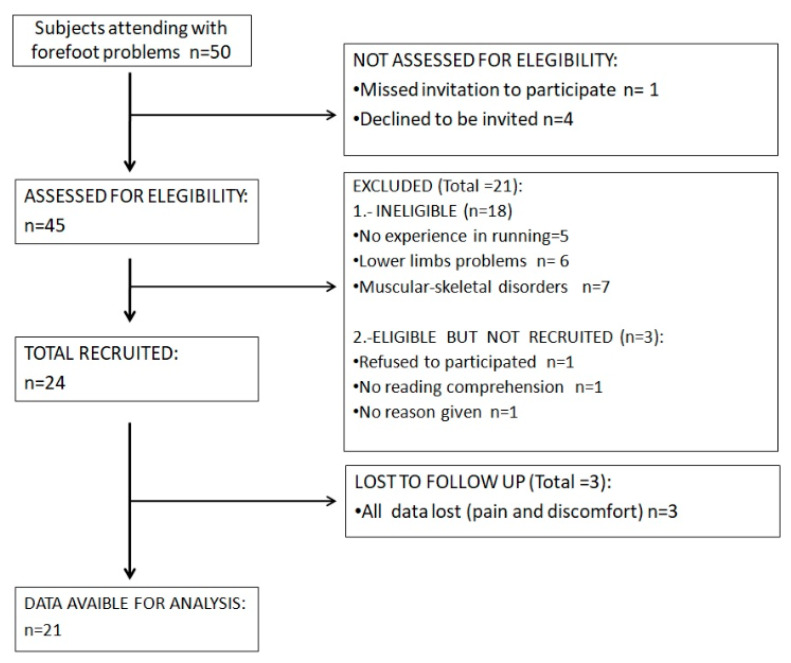
Participant flow chart.

The Shapiro–Wilks test showed a non-normal distribution of the sample (*p* < 0.05), while the Friedman test showed that values were different between the five conditions (*p* < 0.05). All subjects were recruited from a biomechanical clinic in Madrid (Spain) over a two-month period (October to November 2019). Forty-five subjects were asked to participate in the experiment and assessed for eligibility; 24 did not meet the study entry requirements and three withdrew from the study because of pain and discomfort. Ultimately, 21 participants (10 males and 11 females) were enrolled into the study. The participants’ flow chart following the STROBE guidelines, is shown in Figure 4. Sociodemographic data are shown in Table 1.

The reliability of the data obtained from the EMG activity of muscles during running under five different conditions is presented as the ICC and SEM, which are shown in Table 2. Most of the values reached cut-off values over of 0.81 in the ICC data, which suggests “almost perfect reliability” [38], with 0.971 for NIRO-8 mm as the highest value and 0.458 for TMEO-8 mm as the lowest for the gastrocnemius lateralis, and 0.894 for TMEO-8 mm as the highest and 0.767 for NIRO-8 mm as the lowest for the gastrocnemius medialis. Considering the reference that was chosen by the authors, we dismissed TMEO-8 mm values for gastrocnemius lateralis. For SEM, 0.817 mV was the lowest value set for NIRO-8 mm, and 3.766 mV was the lowest value for TMEO-6 mm for the gastrocnemius lateralis, and 2.083 mV was the highest value for NIRO-8 mm and 0.326 mV was the lowest value for TMEO-8 mm for the gastrocnemius medialis. The highest MDC value for TMEO-8 mm was 5.798 mV and 2.264 mV were the lowest value for the gastrocnemius lateralis. Additionally, 5.775 mV was the highest value in the NIRO-8 mm group and 0.904 mV was the lowest value in the TMEO-8 mm group for gastrocnemius medialis.

EMG mean muscle activity in the gastrocnemius medialis and lateralis in SO compared to NIRO-6 mm and 8 mm and TMEO-6 mm and 8 mm are shown in Table 3. In the gastrocnemius lateralis, the EMG activity significantly increased between the SO and NIRO-8 mm (22.27 ± 2.51 vs. 25.96 ± 4.68 mV; *p* < 0.05). There was another statistically significant increase between SO and TMEO-6 mm (22.27 ± 2.51 vs. 24.72 ± 5.08 mV, *p* < 0.05) and vs. TMEO-8 mm (25.49 ± 1.97, *p* < 0.001), but the low ICC of the last value invalidated the reliability of this value. For the gastrocnemius medialis, a statistically significant increase in the EMG activity was noted for SO vs. NIRO-6 mm (22.93 ± 2.1 vs. 26.44 ± 3.63, *p* < 0.001), vs. NIRO-8 mm (28.89 ± 3.6, *p* < 0.001), vs. TMEO-6 mm (25.12 ± 3.51, *p* < 0.05), and vs. TMEO-8 mm (26.38 ± 3.02, *p* < 0.05). The latter was not considered because of its low ICC value. In addition, the relationship between NIROs and TMEOs showed that there was a statistically significant increase in NIRO-6 mm and NIRO-8 mm (26.44 ± 3.63 vs. 28.89 ± 3.6, *p* < 0.05), and a statistically significant decrease in NIRO-8 mm vs. TMEO-6 mm (28.89 ± 3.6 vs. 25.12 ± 3.51, *p* < 0.001) and in NIRO-8 mm vs. TMEO-8 mm (28.89 ± 3.6 vs. 26.38 ± 3.02, *p* < 0.05), although the latter could not be considered because of its low ICC values.

## 4. Discussion

### 4.1. TMEO and NIRO Effects

This is the first study on EMG muscle activity in the gastrocnemius medialis and lateralis under IMTPJ dorsiflexion mobility restrictions by two different kinds of orthoses, the TMEO and the NIRO, in healthy subjects during running. TMEO has been used to treat symptoms of the first stages of OA [9,10,11] moving away dorsally from the contact between the proximal phalanx of the hallux and first metatarsal head surfaces. However, it is unclear if the effects on the triceps surae activity that were caused by the windlass mechanism [24] alteration through the IMTPJ caused the restriction. Some authors have shown the need for proper dorsiflexion of the IMTPJ during the push-off phase to ensure normal activity of the calcaneus–plantar system [24]. We hypothesized that TMEO would increase the EMG triceps surae activity that is induced by restriction of IMTPJ dorsiflexion. Our results showed that EMG activity of the gastrocnemius lateralis and medialis increased with TMEO-6 mm and that there is a further increase with TMEO-8 mm compared to SO (Table 3), although the last one could not be considered because of the low ICC values. Even knowing that there are no studies related to EMG activity during running with the orthopedic restriction of IMPTJ dorsiflexion, these results are consistent with other simulated running research [24,25], which showed that engaging the windlass mechanism by promoting 30° of IMTPJ dorsiflexion caused the arch to absorb and dissipate more elastic energy than under normal circumstances, and likely the energy of the triceps surae would be saved. In the present research, we decreased the windlass capacity through the TMEO, and followed the lack of storage and release energy in the medial longitudinal arch primary in the heel-off phase; this could have been supported by increasing gastrocnemius musculature EMG activity, as shown by our results, and by sustaining the connection between the IMTPJ and triceps surae through the windlass mechanism, according with other authors [24,25].

We hypothesized that NIRO would produce less EMG activity on triceps surae than the TMEO compared to SO. The rationale behind this approach was that its smooth edges and inverted rocker would produce a slight movement restriction of the IMTPJ; therefore, less effort would be required of the triceps surae to move the heel up. However, the present research showed surprising results, with a higher increase in EMG activity in both the gastrocnemius medialis and lateralis muscles (Table 3) with NIRO compared to TMEO, especially with NIRO-8 mm. This could be partly explained because of the soft edges of the NIRO, which yielded instability on the IMTPJ and transferred it to triceps surae in the heel-off phase. This is consistent with other studies with inverted rocker-sole shoes [40] that showed increased plantarflexion at the ankle joint and an increase in lower limb muscular activity [13]. This conclusion is not consistent with other research that showed increasing toe joint stiffness and increased ankle foot push-off work by up to 181% [41].

### 4.2. Osteoarthritis

OA has been defined as one of the most important and incapacitating musculoskeletal disorders in the world and OA of the IMTPJ, is the most commonly affected region on the foot [42]. This pathology can involves partial (FHL) or total (HR) rolling fail of the proximal phalanx of the hallux around first metatarsal bone in the last phase of gait [3], and there are a few treatments to relieve them, looking to avoid contact of the dorsal aspect of theses bones, such as TMEO [9,10,11] or classical rocker soles [12]. No studies about triceps surae EMG activity and IMTPJ OA using orthoses and/or rocker soles during running have been reported; nevertheless, our observations with simulated IMTPJ restriction through TMEO and NIRO, showed an increase of EMG activity pattern of the gastrocnemius medialis and lateralis, in contrast with a recently study [12] with IMTPJ OA and traditional rocker bottom soles, which argued that the reduction of the concentric activity of the triceps surae inferred from the forward displacements of the body center of mass was probably due to passively roll-over of the whole base of support.

### 4.3. Running Economy

Elastic energy is stored and returned by the plantar muscles, plantar aponeurosis, and triceps surae with the Achilles tendon during the mid-stance and heel-off phases of running because of its isometric, concentric, and eccentric stretching–shortening pattern [43,44], which shows that the foot has an important role in RE. RE is related to different biomechanical parameters such as shorter ground contact times, higher stride frequency, joint stiffness, and neuromuscular response [20], specifically the pre-activation of gastrocnemius muscular group [14,17,20]. TMEO and NIRO somehow produced decreased stiffness in the IMTPJ by dorsal migration of the I metatarsal bone, and this was shown by the compensatory increase effect on the gastrocnemius musculature activity that attempts to stabilize IMTPJ instability when joined with the windlass mechanism. This would cause worse RE [20]. Our obtained values confirm the results of some studies [45,46], which showed the importance of neuromuscular pre-activation of the gastrocnemius to increase the leg stiffness, anticipating the loading forces and attenuating the effort of the foot to stabilize the joint as required, improving the energy cost and, therefore, the RE.

## 5. Limitations

The sample size that was calculated in a previous study could not be attained because three individuals were excluded. This must be taken into account when interpreting the results. In addition, we were not able to assess the “order effect” on our sample because didn’t write the different orders of each participant’s choice, despite the fact that both groups had a similar participant number, the hypothetical order effect can take over, and we recommended future study designed to improve this aspect of the assessments. 

Because of the short running test duration when NIRO and TMEO were worn, the hypothetical muscular adaptations of the triceps surae could not be assessed. Longer studies in the future are needed to determine how the exertion levels can influence these muscular adaptations during running.

Considering that most ±SD values obtained in the present research are higher than SEM, authors recommended to have caution in interpreting the results.

## 6. Conclusions

NIRO and TMEO have shown a high interaction with triceps surae, increasing the gastrocnemius medialis and lateralis EMG activity during running. This may be additional evidence of the biomechanics relationship between IMTPJ and the windlass mechanism connection. Higher values of the triceps surae EMG activity wearing NIRO and TMEO during running could have a negative impact on RE; therefore, clinicians should be prescribing them with caution when they want to treat IMTPJ OA in runners.

## Figures and Tables

**Table 1:** Participant demographics.

Variable	*n* = 21 Mean ± SD (95% CI)
**Age**	31.41 ± 4.33 (32.26–35.09)
**FPI (scores)**	3.12 ± 0.17 (2.07–3.41)
**Weight (kg)**	67.50 ± 8.06 (62.36–70.06)
**Height (cm)**	170.08 ± 6.91 (166.9–172.43)
**BMI (kg/m^2^)**	23.15 ± 3.05 (21.7–24.7)

Abbreviations: SD = standard deviation; CI = confidence interval; FPI = foot posture index; and BMI = body mass index.

**Table 2:** Reliability ICC of variables with “shoe only” versus 6- and 8-mm of novel inverted rocker orthoses (NIRO) and traditional Morton extension orthoses (TMEO).

Variables	SO			NIRO-6 mm			NIRO-8 mm			TMEO-6 mm			TMEO-8 mm		
	ICC (95% CI)		MDC	ICC (95% CI)		MDC	ICC (95% CI)		MDC	ICC (95% CI)		MDC	ICC (95% CI)		MDC
		SEM	0.950		SEM	0.950		SEM	0.950		SEM	0.950		SEM	0.950
**Gastrocnemius lateralis (mV)**	0.839			0.932			0.971			0.937			0.458		
	(0.651–0.935)	1.010	3.560	(0.852–0.973)	1.254	3.477	(0.938–0.988)	0.817	2.264	(0.861–0.975)	1.359	3.766	(0.148–0.777)	2.092	5.798
**Gastrocnemius medialis (mV)**	0.848			0.832			0.767			0.872			0.894		
	(0.649–0.94)	0.913	2.530	(0.637–0.931)	1.707	4.731	(0.501–0.905)	2.083	5.775	(0.723–0.948)	1.408	3.904	(0.77–0.957)	0.326	0.904

Abbreviations: ICC = intraclass correlation coefficient; CI = confidence interval; SEM = standard error of measurement; MDC = minimal detectable change; (mV) = millivolts; SO = shoe only; and mm = millimeters.

**Table 3:** Signal amplitudes and comparison values of the mean gastrocnemius lateralis and medialis muscle activities.

	SO	NIRO 6 mm	NIRO 8 mm	TMEO 6 mm	TMEO 8 mm	*p*-Value SO	*p*-Value SO	*p*-Value SO	*p*-Value SO	*p*-Value NIRO 6 mm	*p*-Value NIRO 6 mm	*p*-Value NIRO 6 mm	*p*-Value NIRO 8 mm	*p*-Value NIRO 8 mm	*p*-Value TMEO 6 mm
Variable	mean (mV)	mean(mV)	mean (mV)	mean (mV)	mean (mV)	vs.	vs.	vs.	vs.	vs.	vs.	vs.	vs.	vs.	vs.
**gastrocnemius lateralis**	±SD (95% CI)	±SD (95% CI)	±SD (95% CI)	±SD (95% CI)	±SD (95% CI)	**NIRO 6 mm**	**NIRO 8 mm**	**TMEO 6 mm**	**TMEO 8 mm**	**NIRO 8 mm**	**TMEO 6 mm**	**TMEO 8 mm**	**TMEO 6 mm**	**TMEO 8 mm**	**TMEO 8 mm**
	22.27 ± 2.51	24.65 ± 4.51	25.96 ± 4.68	24.72 ± 5.08	25.49 ± 1.97										
	(20.77–23.279)	(22.41–26.897)	(23.634–28.29)	(23.675–27.35)	(22.19–27.253)	0.085	<0.05 *	<0.05 *	<0.001 **	0.39	0.88	0.356	0.372	0.67	0.913
	22.93 ± 2.1	26.44 ± 3.63	28.89 ± 3.6	25.12 ± 3.51	26.38 ± 3.02										
**gastrocnemius medialis**	(21.88–23.97)	(24.63–28.24)	(27–30.68)	(23.37–26.87)	(24.88–27.89)	<0.001 **	<0.001 **	<0.05 *	<0.05 *	<0.05 *	0.06	0.67	<0.001 **	<0.05 *	0.22

Abbreviations: mV = millivolts; SO = shoe only; NIRO = novel inverted rocker orthoses; TMEO = traditional Morton extension orthoses; mm = millimeters; ±SD = standard deviation; *p* < 0.05 * (95% CI) was considered statistically significant; and *p* < 0.001 ** (95% CI) was considered statistically significant.
